# Insights into the Transcriptional Reprogramming of Peach Leaves Inoculated with *Taphrina deformans*

**DOI:** 10.3390/plants13060861

**Published:** 2024-03-16

**Authors:** Elissaios I. Maniatis, Ioanna Karamichali, Eleni Stefanidou, Anastasia Boutsika, Dimitrios I. Tsitsigiannis, Epaminondas Paplomatas, Panagiotis Madesis, Antonios Zambounis

**Affiliations:** 1 Laboratory of Plant Pathology, Department of Crop Science, Agricultural University of Athens, 11855 Athens, Greece; 2 Laboratory of Agrobiotechnology and Molecular Plant Breeding, Institute of Applied Biosciences (INAB), Center for Research and Technology (CERTH), 57001 Thessaloniki, Greece; 3 Institute of Plant Breeding and Genetic Resources, Hellenic Agricultural Organization Demeter, 57001 Thessaloniki, Greece; 4 Laboratory of Molecular Biology of Plants, Department of Agriculture Crop Production and Rural Environment, School of Agricultural Sciences, University of Thessaly, 38446 Volos, Greece

**Keywords:** biotrophic, DEGs, defense, dimorphism, leaf curl disease, peach, yeast phase

## Abstract

The dimorphic fungus *Taphrina deformans* is the causal agent of peach leaf curl disease, which affects leaves, flowers, and fruits. An RNA-seq approach was employed to gain insights into the transcriptional reprogramming of a peach cultivar during leaf inoculation with the yeast phase of the fungus across a compatible interaction. The results uncovered modulations of specific peach differentially expressed genes (DEGs) in peaches and pathways related to either the induction of host defense responses or pathogen colonization and disease spread. Expression profiles of DEGs were shown to be highly time-dependent and related to the presence of the two forms of the fungal growth, the inoculated yeast form and the later biotrophic phase during mycelial development. In parallel, this differential reprogramming was consistent with a diphasic detection of fungal load in the challenged leaves over the 120 h after inoculation (HAI) period. Leaf defense responses either occurred during the early yeast phase inoculation at 24 HAI, mediated primarily by cell wall modification processes, or more pronouncedly during the biotrophic phase at 72 HAI, as revealed by the activation of DEGs related to pathogen perception, signaling transduction, and secondary metabolism towards restraining further hypha proliferation. On the contrary, the expression patterns of specific DEGs at 120 HAI might further contribute to host susceptibility. These findings will further allow us to elucidate the molecular responses beyond the peach—*T. deformans* interaction.

## 1. Introduction

*Taphrina deformans* (Berk.) Tul. is one widespread fungal pathogen that causes leaf peach curl disease, causing serious economic losses worldwide [1]. The pathogen is a dimorphic ascomycete, which primarily affects the leaves as well as the flowers and fruits of peach orchards in various cultivated regions [2,3]. The most significant symptoms occur from the early spring period when leaves become reddish, severely distorted, wrinkled, and thickened [4]. As a result, tumor-like structures are formed in leaves as well as in fruits due to hyperplasia and the hypertrophy of cells. The reddish areas, upon newly developing leaves, gradually become thicker and curled, turning brown or yellow, and the infected leaves fall prematurely, leading to significant yield losses [5]. 

The fungus in its biotrophic filamentous phase is characterized by septate hyphae and is exclusively found in host tissues as an obligate parasitic form, while its yeast stage is observed in the saprophytic phase [6]. During the initial stages of infection, *T. deformans* enters the host cells through the stomata and stimulates host cell growth and division [7]. Even though the exact mechanisms concerning tumour development are still unclear, it was proposed that the infection causes a hormonal imbalance in the host, leading to the formation of tumours as *T. deformans* synthetizes and excretes auxins and cytokinins [8]. Modifications in the anatomical structure of the host cells have also been observed during the infection [9]. Polysaccharide-degrading enzymes, such as cellulase, are secreted by the growing hyphae in the intercellular spaces, causing the host cell walls to be partially dissolute. Thus, the plant plasma membrane is modified, resulting in vesiculation, and the plant middle lamella is dissolved [10]. Unlike other fungi, *T. deformans* does not form haustoria; instead, the pathogen forms a close nutritional relationship with the host cell wall and plasma membrane complex to obtain nutrients [10].

In contrary with other biotrophic and dimorphic fungi, such as *Ustilago maydis*, the transition to the biotrophic phase and the induction of *T. deformans* dimorphism is quite slow. As a result, only two days after infection did the *T. deformans* yeast cells turn into their filamentous form, whereas the majority of the *U. maydis* haploid sporidia were able to infect maize after 12 HAI by forming hyphae and developing appressoria [11]. Furthermore, in the peach–*T. deformans* pathosystem, early host responses are not observed earlier than around 12 h after infection [11], while it was reported that the filamentous form of *T. deformans* is adequately developed in peach leaves after inoculation with blastospores in the yeast phase of the pathogen [7]. The identification of peach genes and pathways conferring tolerance to the fungus has proven to be a challenging task because of the complex molecular interactions that occur between the pathogen and the host cells in the apoplast [7]. In parallel, resistance to *T. deformans* is rare because very few or none of the used cultivars have been identified as tolerant through peach genotype screenings [12,13].

RNA-sequencing (RNA-seq) technology has been widely adopted to provide valuable insights into plant–pathogen interactions, allowing for the accurate capture of host transcriptomic responses during infection with pathogens [14]. Thus, hundreds of transcriptome profiling studies have been conducted to date, establishing RNA-seq as a sophisticated approach to unravel the molecular mechanisms of such interactions [14]. Furthermore, since the initial introduction of next-generation sequencing (NGS) technology in 2006, the recent advances of this technology allow the researchers to adopt fast and high-throughput transcriptomic approaches to decipher the mechanisms behind altered gene expression during plant–pathogen interactions [15]. Nowadays, sequencing costs continue to decrease, and RNA-seq technology is becoming more feasible by employing prominent NGS platforms, such as those of Illumina and SMRT (Single-molecule real-time) [15,16,17].

In the present study, we uncovered the leaf transcriptome profiles of a sensitive cultivar after its inoculation with the yeast phase of *T. deformans*. We identify key genes and pathways involved in the pathogenesis as well as in the induction of some extent of host defense responses during this interaction. To the best of our knowledge, this is the first large-scale transcriptome analysis in the peach–*T. deformans* pathosystem. These findings will contribute to our deeper understanding of the molecular interplay beyond this interaction and could lead to the establishment of novel crop protection strategies towards mitigating the impact of *T. deformans* on peach crops. 

## 2. Results

### 2.1. T. deformans Leaf Inoculation and Monitoring of Pathogen Growth

**Figure 1:**
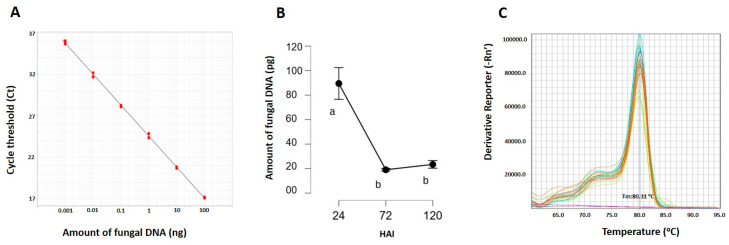
(**A**) Standard curve constructed using six 10-fold serial dilutions (red circles) ranging from 100 ng up to 1 pg of fungal DNA extracted from pure *T. deformans* culture (strain TdLar1). Three replicates were performed for each fungal DNA dilution (R^2^: 0.969, Eff.: 90.238). (**B**) The fungal DNA quantity (ng) of *T. deformans* (strain TdLar1) that was present in 10 ng of total leaf DNA extracted from artificially inoculated leaves (TD) of Andross cultivar at 24, 72, and 120 h after inoculation (HAI). The vertical bars represent the least significant difference (Tukey test, significance 99%) using three biological replicates. Different letters below the vertical bars indicate statistically significant differences (*p* < 0.05). (**C**) A calibration melting curve plot showing the sensitivity and robustness of *T. deformans* quantification based upon the profiles of qPCR curves for fungal DNA that was either present in 10 ng of total leaf DNA extracted from artificially inoculated leaves (TD) of Andross cultivar at 24, 72, and 120 HAI or was extracted from a pure pathogen culture using six 10-fold serial dilutions. In all reactions, a specific Tm peak at 80.31 °C was observed.

*T. deformans* blastospores (yeast phase) of the strain TdLar1 were used for the leaf inoculation of a sensitive peach cultivar (cv. Andross). During this compatible interaction, a diagnostic assay was developed to confirm and monitor the amount of fungal DNA in the inoculated leaves, tracking the progress of infection during the various stages of fungal colonization over a 120 h period. There were no macroscopic differences between the leaves inoculated with *T. deformans* and the mock-inoculated leaves during this pre-symptomatic period of infection. The RT-qPCR assay relied on the specific amplification of a fragment of the *T. deformans Tub2* gene using a pair of species-specific primers. Initially, the sensitivity and reliability of the diagnostic assay was validated by performing six 10-fold serial dilutions of fungal DNA ranging from 100 ng up to 1 pg. A standard curve was constructed, and the assay was able to successfully detect quantities down to 1 pg of *T. deformans* DNA (Figure 1A). Then, in order to measure the fungal growth in planta, the total DNA was extracted from both the *T. deformans* inoculated leaves (TD) and the mock-inoculated leaves (CT) across the three time points upon the challenge with the pathogen blastopores. The designed primers (TdbtqF and TdbtqR), which target the partial coding sequences of *T. deformans* beta-tubulin encoding gene, were used to successfully detect and quantify the pathogen exclusively in artificially inoculated samples. Particularly, a biphasic curve was revealed, reaching its peak early at 24 HAI when the fungus was still in the yeast form, declining at 72 HAI and then slightly rising again at 120 HAI afterwards when the pathogen presumably assumed the filamentous form (Figure 1B). The sensitivity and robustness of the detection was not affected by the equal inclusion of 10 ng of peach genomic DNA in the qPCR assays. A calibration melting curve plot was constructed, confirming the sensitivity and robustness of all the diagnostical assays (Figure 1C). 

### 2.2. Physiological Changes in Leaves in Response to T. deformans Inoculation

**Figure 2:**
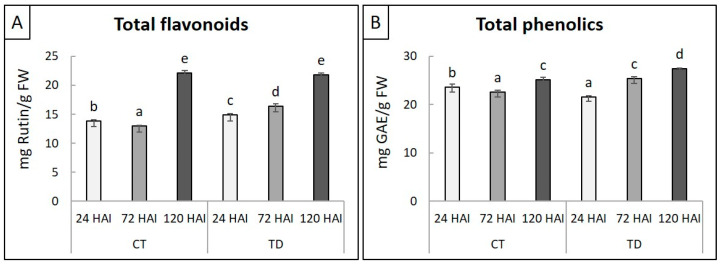
Physiological indicators of peach leaves between *T. deformans* inoculated (TD) and mock-inoculated (CT) samples across the three time points. (**A**) Total flavonoids, (**B**) Total phenolics. Bars indicate the mean values of three biological replicates ± standard deviations. Statistical analysis was performed using one-way ANOVA followed by Tukey’s multiple comparison post hoc test. Different letters represent statistically significant differences (*p* < 0.05) between treatments across the different time points.

For the examination of the physiological responses associated with the *T. deformans*–peach interaction, total flavonoid and phenolic compounds were evaluated in leaves challenged with *T. deformans* or mock inoculated across the three time point series. The amount of flavonoid compounds in TD treatments were progressively and significantly increased at 24 and 72 HAI in comparison to CT treatments, reaching their highest amount at 120 HAI (Figure 2A). In contrast, the amount of total phenolics was statistically significantly higher at 72 and 120 HAI in the TD treatments compared to the CT treatments. However, the concentration of phenolic compounds in the TD treatment exhibited a significant decrease in comparison to the CT treatment at 24 HAI (Figure 2B).

### 2.3. RNA-Seq Analysis of Leaves after Inoculation with T. deformans

**Figure 3:**
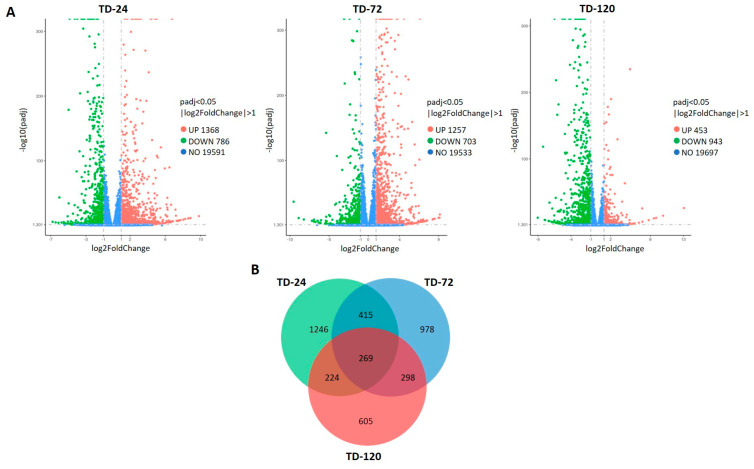
(**A**) Volcano plots of differential expressed genes across the three comparison groups (TD-24, TD-72, TD-120). The green part represents downregulated genes and the red part represents up-regulated genes. No significantly expressed genes are represented in blue. The *x*-axis represents the log2foldchange and the *y*-axis the base mean expression values. (**B**) Venn diagram representing the distribution of DEGs across the three comparison groups (TD-24, TD-72, and TD-120).

In order to gain insights into the peach transcriptomic responses during *T. deformans* inoculation, we report, for the first time, an RNA-seq approach. A detached leaf assay was performed, and leaf samples were either inoculated with *T. deformans* (TD) or mock inoculated (CT). Leaves were collected from a sensitive cultivar and inoculated with *T. deformans* in its yeast phase using a blastospore suspension, and the transcriptional reprogramming was monitored across three time series during a 120 h period that covered the progress of infection. To study the changes occurring across the three time points (24, 72, and 120 HAI), eighteen sequencing libraries were constructed, and a total of 944,341,404 high-quality pair-end reads were generated. For each sample approximately 93.21% of the reads could be mapped to the reference peach genome, while 88.79% of them matched uniquely (Table S1). The DEGs between TD versus CT samples were assigned in three comparison groups named TD-24, TD-72, and TD-120 across the three time points with a threshold of log2foldchange ≥1. The expression patterns of the RNA-seq data suggest dynamic and time-dependent transcriptional reprogramming upon infection. A total of 5510 DEGs were detected, with 2154, 1960, and 1396 DEGs identified in TD-24, TD-72, and TD-120 comparison groups, respectively (Table S2). The majority of them were upregulated, except at the TD-120 group, where the downregulated DEGs were more than the upregulated ones (Figure 3A). In addition, among these DEGs, 1246, 978, and 605 were specifically induced at 24, 72, and 120 HAI, respectively, whereas 269 DEGs were constitutively co-expressed across all the time points (Figure 3B). 

### 2.4. Functional Annotations and Classifications of Peach DEGs

For the evaluation of potential functions of DEGs, they were assigned to significant annotations upon a GO term enrichment analysis and classified according to their molecular function, cellular component, and biological process. Across the three time points, DEGs assigned to GO terms corresponding to molecular functions, such as “DNA binding transcription factor activity”, “transcription regulator activity”, “xyloglucan:xyloglucosyl transferase activity”, and “oxidoreductase activity, acting on paired donors”, along with these related to “cell wall” cellular components, were constitutively induced. Particularly, several DEGs assigned to the above GO terms were mostly upregulated at 72 HAI while being suppressed at the late time point. Notably, the GO term “defense response” of biological processes was a significantly enriched group and overrepresented no earlier than 72 HAI, while the majority of assigned DEGs were upregulated at 72 HAI and down-regulated at 120 HAI (Figure S1; Table S2).

**Figure 4:**
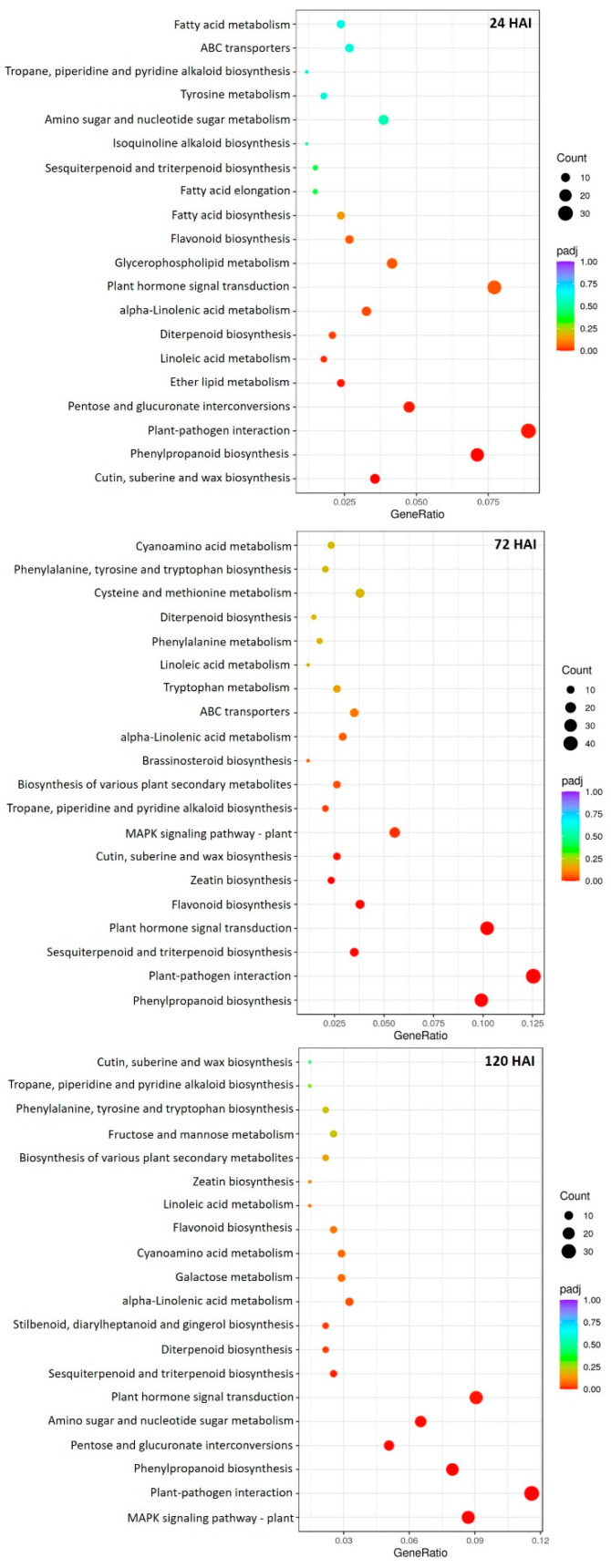
Dot plots showing the KEGG enriched pathways of DEGs identified across the three comparison groups (TD-24, TD-72, and TD-120).

Following a KEGG pathway analysis, the DEGs were further assigned to 20 of the most enriched categories to identify the involved pathways in the peach transcriptome upon *T. deformans* inoculation. Among them, a significant induction of DEGs related to the “plant–pathogen interaction” pathway was observed; this pathway was the most highly abundant KEGG pathway across all time points. Notably, the portion of DEGs assigned to this pathway was mostly more upregulated at 72 HAI. At the same time point, DEGs related to “biosynthesis of various plant secondary metabolites” “MAPK signaling pathway”, “phenylpropanoid biosynthesis”, “plant hormone signal transduction”, “sesquiterpenoid and triterpenoid biosynthesis”, and “zeatin biosynthesis” were mostly upregulated. On the contrary, mainly at 120 HAI, the majority of DEGs associated with the above significant enriched pathways were mainly suppressed. In addition, significant enriched pathways, such as “cutin, suberine and wax biosynthesis”, “linoleic acid metabolism”, and “alpha-linolenic acid metabolism”, were observed at 24 HAI, and numerous related DEGs were up-regulated. Worth mentioning is the “flavonoid biosynthesis” pathway’s constitutive enrichment at 24 and 72 HAI (Figure 4; Table S2).

### 2.5. DEGs Involved in Cell Wall Modification and Degradation

**Figure 5:**
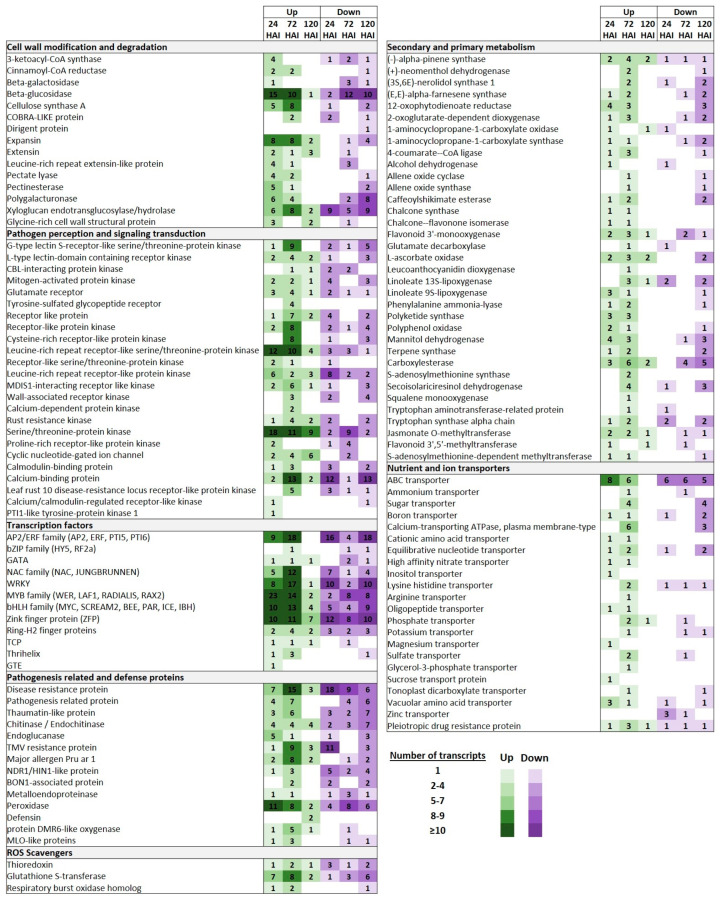
Selection of key DEGs upregulated (Up) and downregulated (Down) in peach leaves across the three comparison groups (TD-24, TD-72, and TD-120) based on the three time points upon inoculation with *T. deformans* (24, 72, and 120 HAI). To each gene category, the numbers of the differentially expressed transcripts are reported.

The cell wall constitutes a physical structural barrier at sites of infection that restricts the fungal spread. In our study, a relative abundant inventory of DEGs involved in various cell wall modification and degradation processes in the epidermis of inoculated leaves were observed upon *T. deformans* inoculation even at the early yeast phase. Thus, several DEGs, such as those encoding 3-ketoacyl-CoA synthase (KCAs), beta-glucosidase (GL), cellulose synthase (CesA), expansin (EXP), extensin containing leucine-rich repeat (LRR-EXT), pectate lyase (PL), polygalacturonase (PG), pectinesterase (PME), and glycine-rich cell wall structural protein (GRP) proteins, were mainly upregulated. This expression pattern was also maintained, albeit to a lesser extent, at 72 HAI. On the other hand, at 120 HAI, the expression profile of these genes underwent a significant alteration with the majority of them, along with nine members of the XTH (xyloglucan endotransglucosylase/hydrolase) family and a dirigent protein gene, suppressed (Figure 5; Table S3).

### 2.6. DEGs Involved in Pathogen Perception and Signaling Transduction

Numerous DEGs encoding pathogen recognition receptors (*PRRs*) were significantly induced, mainly at 72 HAI, including various types of receptor-like kinases (RLKs) and receptor-like proteins (RLPs). This suggests that pattern-triggering immunity (PTI) is a quite conserved defense strategy in peaches upon being challenged with *T. deformans*. Thus, several DEGs encoding receptors containing lectin domains (LecRKs), such as the G-type LecRKs and L-type LecRKs, CRKs (cysteine-rich receptor-like kinases), WAKs (wall-associated receptor kinases), GRs (glutamate receptors), MDIS1-interacting receptor like kinases, and members of LRR receptor-like serine/threonine-protein kinase family (LRR-RLKs), were mainly upregulated. Among the DEGs involved in signaling transduction, it is worth mentioning the high upregulation of genes encoding calcium-binding proteins (CaBPs) at the TD-72 comparison group. Notably, at 24 HAI, the number of suppressed DEGs involved in pathogen perception and downstream defense-signaling cascades was more abundant compared to those that were downregulated at 72 HAI, with the exception of those encoding serine/threonine protein kinases (STPKs). Finally, at the late time point, the induced DEGs involved in signaling transduction were mostly suppressed, although in the cases of *STPKs*, *LRR-RLKs*, and cyclic nucleotide-gated ion channel (CNGC)-encoding genes, the majority of them were upregulated (Figure 5; Table S4).

### 2.7. DEGs Encoding TFs

A wide range of TF-encoding genes belonging to different families (ERFs, WRKYs, MYBs, NACs, bHLHs, BZIPs, ZFPs, and HSFs) were mostly upregulated at 48 HAI and were downregulated at the late time point. Notably, at the TD-24 comparison group, sixteen members of *ERFs* were suppressed, while 23 members of the MYB family were significantly upregulated (Figure 5; Table S5).

### 2.8. DEGs Encoding Pathogenesis-Related and Defense Proteins

The regulation of PRs and defense proteins, mainly at 72 HAI, further indicates the induction of immunity responses on peach leaves upon challenging with *T. deformans*. Particularly, the RNA-seq data revealed that a range of DEGs encoding disease resistance proteins, such as members of defense proteins of NHL family (NDR1/HIN1), TMV resistance proteins, along with various classes of PRs, such as endoglucanase (PR-2), chitinase/endochitinase (PR-3), thaumatin (PR-5), and major allergen Pru ar 1 (PR-10) proteins, were mainly upregulated at this time point. Notably, DEGs encoding BON1-associated proteins (BAPs) were also highly upregulated at the TD-72 group. In the contrary, eighteen and eleven DEGs encoding disease resistance proteins and members of TMV resistance proteins were suppressed, respectively, at 24 HAI. However, at 24 HAI, eleven peroxidase (PR-9)-encoding genes were upregulated, while the majority of the pathogenesis-related and defense proteins were suppressed at 120 HAI (Figure 5; Table S6).

### 2.9. DEGs Involved in Secondary and Primary Metabolism

DEGs involved in phenylpropanoid biosynthesis, such as those encoding 4-coumarate–CoA ligase (4CL) and phenylalanine ammonia-lyase (PAL), as well as in flavonoid biosynthesis, such as those encoding chalcone synthase (CHS), chalcone-flavonone isomerase (CHI), leucoanthocyanidin dioxygenase (LDOX), and polyketide synthase (PKS), were significantly upregulated at 24 and 72 HAI. Similarly, DEGs involved in sesquiterpenoid and triterpenoid biosynthesis, such as those encoding squalene monooxygenase and alpha-pinene synthase, were mainly activated at 72 HAI. On the contrary, at 120 HAI, the majority of DEGs involved in secondary and primary metabolism were downregulated. Furthermore, through the upregulation of genes encoding 12-oxophytodienoate reductase (OPR), allene oxide cyclase (AOC), allene oxide synthase (AOS), and various types of linoleate lipoxygenase (LOX) proteins, the pathway of “alpha-linolenic acid metabolism” involved in primary metabolism was enriched mostly at 72 HAI (Figure 5; Table S7).

### 2.10. DEGs Encoding Nutrient and Ion Transporters

Several different types of DEGs encoding transporters were found to be significantly induced; most of these genes were upregulated at 72 HAI, downregulated at 120 HAI, and mostly upregulated at 24 HAI. For example, six and four DEGs encoding calcium-transporting ATPases and sugar transporters were upregulated, respectively, at 72 HAI. On the other hand, five and four ABC and sugar transporters were downregulated, respectively, at 120 HAI. Furthermore, various oligopeptide, potassium, phosphate, and amino acid transporters were also induced across the three comparison groups (Figure 5; Table S8).

### 2.11. Validation of RNA-Seq Data Using qRT-PCR

The relative expression values from the RNA-seq analysis were validated using a qRT-PCR assay. A set of nine DEGs were randomly selected to validate the transcriptomic results, and the gene-specific primers are listed in Table S9. Seven genes showed similar expression patterns (log2foldchange values) to those of the RNA-Seq (Figure S2). 

## 3. Discussion

*T. deformans*, the causal agent of peach leaf curl disease, is a dimorphic fungal pathogen that causes significant economic losses globally [5,7]. The fungus is considered an obligate parasite as the filamentous form develops stable hyphae exclusively in the host tissue. Even though nearly all commercial cultivars are susceptible to the pathogen, the transcriptional responses of peaches upon leaf inoculation with *T. deformans* remain widely unknown. The aim of this study was to decipher the transcriptome dynamics and the regulatory mechanisms underlying this interaction during the pre-symptomatic stage of the disease. Thus, a three time-series period was employed to cover diverse stages of infection, as previously it was reported that the transition of *T. deformans* from the yeast to the filamentous form took place no earlier than 48 HAI [7]. Since it was previously reported that the transition of *T. deformans* from the yeast to the filamentous form occurred no earlier than 48 HAI [7], a three time-series period was employed to cover diverse stages of infection in order to gain insights into transcriptional responses beyond challenges with the pathogen. 

Furthermore, a sensitive and reliable qPCR assay was developed for tracking the quantity of fungal DNA associated with its colonization, and two physiological indicators were monitored in the inoculated peach leaves. The quantification and detection threshold of the pathogen was achieved with qPCR using newly designed species-specific primers targeting the tubulin gene of *T. deformans.* The designed primers (TdbtqF and TdbtqR) were used to successfully detect and quantify *T. deformans* in artificially inoculated leaves, where the absence of leaf hyperplasia indicated that symptoms had not yet been developed during the 120 HAI period. A biphasic curve of the fungal detective amount was remarkably revealed by the leaf diagnostic assay. It peaked early at 24 HAI when the fungus was still in the yeast form, declined at 72 HAI, and then slightly increased again at 120 HAI when the pathogen most likely assumed the filamentous form. This detective profile seems to follow the dimorphic nature of the fungus. Notably, peach leaf tissues of sensitive genotypes also underwent a similar biphasic metabolomic reprogramming upon inoculation with *T. deformans* blastospores (yeast phase), with an initial stage coinciding with the yeast form and a second stage occurring when the hyphae were developed [7].

Our RNA-seq data revealed that early responses at 24 HAI are partially activated in peach leaves in response to *T. deformans* inoculation with its yeast phase. This dynamic reprogramming is also in line with the dimorphic lifestyle of the pathogen. The significant enrichment of the “cutin, suberine and wax biosynthesis” pathway indicates the activation of numerous DEGs involved in cell wall degradation and modification processes, as was also previously reported during the early yeast phase of the pathogen inoculation [7]. The upregulation of a number of *KCA*, *CesA*, *LRR-EXT*, and *GRP* genes at 24 HAI is associated with hampering the pathogen penetration by promoting lignin-mediated cell wall reinforcement, the stiffening and thickening of cell walls [18,19,20,21]. In contrast, the constitutively upregulation of *EXP*, *PL*, and *PG* genes that influence cell wall extensibility and promote susceptibility [22,23] might contribute to pathogen colonization during the inoculated yeast form at 24 HAI. Another putative susceptibility factor that could be considered at early responses is the activation of 15 *GL* genes, which may be directly manipulated by the pathogen. It is also known that pectin is directly targeted by fungal cell wall-degrading enzymes (CWDEs), potentially facilitating pathogen colonization. Thus, the activation of *PME* genes associated with cell wall degradation potentially played a role in further promoting *T. deformans* pathogenesis at 24 HAI. Overall, these results indicate that cell wall modification processes were activated in response to *T. deformans* inoculation at 24 HAI; however, they would be inefficient to further block the *T. deformans* penetration into the leaf tissue. After the *T. deformans* transition from the yeast to the filamentous form at 72 HAI, the expression profiles of DEGs involved in cell wall degradation and modification processes were slightly altered; for example, we observed a significant downregulation of twelve *GL* genes along with an upregulation of ten similar DEGs. This suggests a transition to the partial activation of cell wall-related defense responses, in constituency with both the diphasic alteration in the amount of pathogen detected in the diagnostical assay and the lifestyle of *T. deformans.* Finally, at 120 HAI, the expression profile of cell wall-related DEGs was significantly altered, with most of them to being suppressed. The simultaneous suppression of adequate members of *XTHs* as well as of a *dirigent* and a COBRA-like protein gene further indicates the promotion of susceptibility during *T. deformans* mycelial development.

The high upregulation of DEGs, such as those of *G-*type *LecRKs*, *L-*type *LecRKs*, *CRKs RLKs*, and *WAKs*, involved in pathogen perception and signal transduction, mainly at 72 HAI, indicates that peach leaves actually recognize *T. deformans* during its biotrophic form in the mycelial development, most probably through the so-called pathogen-associated molecular patterns by activating of various *PRRs* [7,24]. Among the induced *RLKs*, the *G-*type *LecRK* and *WAK* genes are known to be involved in plant defense and pathogen recognition [19,21,25,26,27,28]. Notably, even at 24 HAI, when the fungus is still in its inoculated yeast form, 12 *LRR-STK* and 18 *STPK* genes were upregulated, highlighting the important triggering role of PTI participation in the early defense responses upon inoculation. It is worth mentioning that the expression profiles of a range of induced *PRR* genes, such as those of *WAKs* and *CRKs*, follow a biphasic pattern during the 120 period, which likely correspond to the presence of either the inoculated yeast or the later biotrophic phase of the pathogen. Finally, the significant suppression of DEGs involved in pathogen perception and signaling transduction of immune cascades at 120 HAI, such as in the case of genes encoding CRKs, WAKs, G-type LecRKs, MDIS1-interacting receptor line kinases, and MAPKs, allow us to speculate that any transcriptional reprogramming towards the establishment of PTI was interrupted, perhaps though its manipulation by *T. deformans.*

Defense mechanisms against pathogen infections are regulated by various families of TFs [19,29,30]. In our study, the abundant inventory of activated *TFs* around the 120 HAI period was confirmed in the presence of both forms of the fungus. Particularly, TF-encoding genes were mainly upregulated at 72 HAI, mostly those of NAC, WRKY, MYB, and bHLH families, which may contribute to the activation of defense reactions simultaneously with the fungal load decreasing. Among them, it is known that *WRKY33* regulates the biosynthesis of camalexin and induces the expression of defense-related genes by activating JA-dependent processes [27]. Furthermore, members of the bHLH family regulate JA-mediated overlapping signaling pathways [31], whereas members of ZFP family mediate metabolic modulations towards the establishment of disease responses [32]. Furthermore, TFs of bHLH and ZFP families were significantly upregulated at 72 HAI. However, it is interesting to note that, at 72 HAI, the significant upregulation of 18 members of the AP2/ERF family, which regulate molecular responses against pathogenic fungi by integrating signals involving the ethylene hormone, may contribute to a condition that is conducive to susceptibility [18]. Despite the high induction of TFs at 72 HAI, a range of TFs were also induced during the yeast phase inoculation stage, such as members of the MYB family, which modulate complex defense networks, signaling pathways, the induction of flavonoid biosynthesis, and early PTI activation upon fungal attack [33]. The majority of TFs were suppressed at 120 HAI, indicating that any defense reactions revealed at 72 HAI were repressed at this time point, as *T. deformans* had effectively colonized and spread throughout the filamentous and biotrophic phases on peach leaves. Previously, it was postulated that JA-dependent, defense-related pathways were activated in susceptible genotypes upon *T. deformans* challenge [7]. In this line, our results also highlight the JA as the main phytohormone participating in signaling transduction-mediated responses, as several JA biosynthesis-related genes, such as *OPRs*, *AOCs*, *ACC oxidases*, and *lipoxygenase* genes, were upregulated, mainly at 72 HAI.

The higher upregulation of PRs and defense-related DEGs, mainly at 72 HAI, indicates a delay in inducible immunity responses that occur no earlier than the transition from yeast to biotrophic phase upon inoculation. Previously, it was reported that the production of PR proteins is triggered upon *T. deformans* leaf inoculation in both susceptible and resistant peach genotypes [7]. In our study, a considerable number of DEGs encoding peroxidase and endoglucanase genes were remarkably upregulated at 24 HAI, suggesting that a defense response was induced even during yeast phase inoculation, although to a lesser extent than in the biotrophic phase, which was unable to restrict *T. deformans* growth. In contrast, at 120 HAI, a pronounced tendency of repression upon the majority of pathogenesis-related and defense genes was observed, which is consistent with the previously reported restoration of normal levels of such transcripts after early peaks of induction in susceptible genotypes [7]. A similar suppression of early response genes after their initial activation was reported in maize after infection with the biotrophic fungi *U. maydis* [11], while the hemibiotrophic fungus *Colletotrichum graminicola* does not promote any host defenses during the biotrophic phase [34]. In our study, the activation of members of the PR-10 family at 72 HAI, such as DEGs encoding major allergen Pru ar 1 proteins, suggests a further enhancement of the JA-mediated transduction of defense signaling, as it is known that PR-10 proteins control the JA-biosynthesis pathway by interacting with metabolic components of flavonoid biosynthesis [35]. These findings are in agreement with [7], while a similar expression pattern was revealed at 72 HAI regarding other plant defense regulators with significant roles in the immune signaling hub, such as DEGs encoding various disease and TMV resistance proteins. Furthermore, two DEGs encoding orthologs of BON1-associated proteins were upregulated at 72 HAI, while they were suppressed at 24 and 120 HAI, revealing a biphasic profile in consistency with the diphasic detection of fungal loads in our study. Thaumatin-encoding DEGs were also mainly upregulated at 72 HAI, while they were suppressed at 120 HAI. Previously, the induction of a thaumatin gene in susceptible genotypes upon challenge with *T. deformans* was reported [7]. Notably, five DEGs encoding members of DMR6-like oxygenases, which are known negative regulators of immunity [36], acting as sensitivity factors of basal defense responses, were up-regulated at 72 HAI, suggesting that defense responses were suppressed to some extent, contributing to *T. deformans* susceptibility. Previously, researchers postulated the lack of any inducible *defensin* (*DFN*)-encoding genes in sensitive genotypes upon their challenge with *T. deformans*, which was correlated with susceptibility to the fungus [7]. It is worth mentioning that, in our study and in contrast with the overall tendency of the suppression of defense-related genes at 120 HAI, two *DFN* genes were exclusively upregulated at this stage, suggesting that defense responses were also present, even in a lesser extent, at 120 HAI.

The secondary metabolic shunt was not revealed earlier than the transition to the biotrophic phase, as the higher abundance of upregulated DEGs linked to the biosynthesis of secondary metabolites was mostly observed at 72 HAI and partially at 24 HAI. On the contrary, at 120 HAI, a remarkable suppression of DEGs involved in the biosynthesis of secondary metabolites was evident. Furthermore, this differential modulation of secondary metabolism is consistent with the diphasic detection of a fungal load, which might perturb metabolism to some extent. These biphasic responses over the 120 HAI period, which may be related to the presence of both forms of the fungus, are consistent with the metabolomic profiles of sensitives genotypes after inoculation with *T. deformans* yeast form [7]. Particularly, in our case, this delayed activation of related DEGs at 72 HAI is signified by the highly enriched KEGG terms “biosynthesis of various plant secondary metabolites”, “phenylpropanoid biosynthesis”, “sesquiterpenoid and triterpenoid biosynthesis”, and “zeatin biosynthesis”, mainly at this time point. In addition, the concentration of total phenolic compounds was significantly higher at the *T. deformans*-inoculated leaves in comparison with the mock-inoculated leaves at 72 and 120 HAI, progressively increased up to 120 HAI, implying their important role in *T. deformans* pathogenicity. Even though the induction of the phenylpropanoid biosynthesis pathway is crucial for phenol biosynthesis and the initiation of basal defense responses, it seems that it was unable to restrict fungal growth. Furthermore, the constitutive enrichment of the “flavonoid biosynthesis” KEGG pathway across the three time points is also likely correlated with the activation of defense responses, mainly at 24 and 72 HAI; previously, flavonoid polyphenols were correlated with the competence of unripe strawberries to restrict the growth of *Botrytis cinerea* [27]. In accordance, the analysis of total flavonoids revealed that their concentrations were significantly higher in leaves challenged with the fungus than from the mock-inoculated leaves at 24 and 72 HAI. 

It should be noted that a high enrichment of various primary biosynthetic KEGG pathways, including that of tryptophan metabolism, was revealed at 72 HAI. Previously, the concentration of tryptophane metabolite was found to be induced in susceptible genotypes upon *T. deformans* inoculation [7]. Among the different roles in the pathogenesis that this metabolite is involved in, it constitutes the precursor for the synthesis of auxin [7]. Considering that the symptoms of the disease (hyperplasia and tissue curling) have been linked to a leaf hormonal imbalance caused by the fungus [7], the induction of tryptophan metabolism in our study may facilitate the induced synthesis of auxins during the pre-symptomatic stages of the disease. Apart from this, *T. deformans* appears to manipulate plant cell metabolism in susceptible genotypes, as it has already been described in other pathosystems involving biotrophic fungi [7]. Thus, the induction of DEGs linked to amino acids biosynthesis, which was observed in our study with the enrichment of related KEGG pathways, might serve either to provide a nitrogen source for the pathogen, promoting further pathogenesis, or it might be manipulated earlier by *T. deformans* for the induction of its dimorphic transition. On the other hand, this reprogramming of primary metabolism may activate peach defense responses by providing extra precursors for the biosynthesis of secondary metabolites during infection, as it was reported in *Botrytis* infections [21,37]. Furthermore, KEGG pathways related to linolenic acid metabolism was significantly enriched at 72 HAI through the upregulation of *OPR*, *AOC*, *AOS*, and *LOX* genes reinforcing the hypothesis that their activation is a component of a defense strategy upon *T. deformans* challenge.

In our study, a range of nutrient and ion transporter-encoding DEGs were upregulated, mainly at 72 HAI and to a lesser extent at 24 HAI, while at 120 HAI, they were almost suppressed. It was previously postulated that several of them might have been manipulated by pathogens to enable nutrient uptake from decayed host cells for their growth and development during the early stages of colonization [38]. Among those worth discussing is the constitutive upregulation at 72 HAI of sugar transporter- and calcium-transporting ATPase-encoding DEGs. As it was previously postulated during its biotrophic phase, *T. deformans* establishes a dynamic relationship with host cells in order to transfer and obtain the required nutrients for hyphae development and reproduction [7]. Furthermore, a crucial virulence factor necessary for the biotrophic growth of *U. maydis* is the induction of a high-affinity sucrose transporter during filament and appressorium formation [39].

In conclusion, we report a high dynamic transcriptional reprogramming during the *T. deformans* interaction after leaf inoculation with the yeast phase of the fungus in a sensitive peach genotype. The expression profiles of DEGs seem to be highly time dependent and related to the presence of the two forms of the fungus, the inoculated yeast form and the later biotrophic phase during mycelial development. Apart from the relation of DEG expression profiles to the dimorphism transition of *T. deformans*, this differential modulation is consistent with the diphasic detection of fungal loads in challenged peach leaves, employing a new diagnostical assay. In order to prevent the progression of the disease, any leaf defense responses occur either during the early yeast phase inoculation at 24 HAI, mediated mainly by cell wall modification processes, or more pronouncedly during the biotrophic phase at 72 HAI, as revealed by the activation of DEGs related to pathogen perception, signaling transduction, and secondary metabolism to restrain further hypha proliferation. On the contrary, the expression patterns of specific DEGs at 120 HAI further contribute to peach susceptibility. 

## 4. Materials and Methods

### 4.1. Pathogen Isolation

*T. deformans* strain TdLar1 was isolated from a naturally infected leaf from a peach (*Prunus persica* L. cv Andross) orchard in the Larisa region (Central Greece). The leaf displayed typical disease symptoms, including severe distortion, red coloration, wrinkles, and thickening. The pathogen isolation was performed using the spore fall method [40] with minor modifications. Briefly, after the validation of the characteristic asci with microscopical observation, circular leaf discs (0.5 cm in diameter) of the infected leaf blade were attached on a petri dish cover. The cover was used to close a Petri dish filled with PDA (potato dextrose agar), supplemented with 50 μg/mL streptomycin, and the bursting *T. deformans* asci released ascospores onto PDA substrate. A blastospore colony (yeast phase) was produced by the germination of the ascospores and was used for streaking on YMA medium [0.3% (*w*/*v*) yeast extract, 0.5% (*w*/*v*) peptone, 1% (*w*/*v*) glucose, 0.3% (*w*/*v*) malt extract, and 1.5% (*w*/*v*) agar, pH 5–6] supplemented with streptomycin. A single cell colony was then transferred to yeast malt agar (YMA) and was kept in a growth chamber at 22 °C. The pathogen was microscopically and molecularly identified as *T. deformans* (NCBI accession OR888756) and stored as glycerol stock at −80 °C.

### 4.2. Plant Material and T. deformans Inoculation

Peach seedlings (cv. Andross) were grown in the greenhouse at 25 °C with 60–70% relative humidity and 16/8 h photoperiod. Young and healthy leaves with no visible symptoms of *T. deformans* infection were gently detached and inoculated using a blastospore suspension (9 × 10^6^ spores mL^−1^) of the yeast phase of TdLar1 strain by diluting a 4-day-old yeast phase culture in YMB liquid medium. For each leaf, the abaxial surface was inoculated with approximately 120 μL blastospore suspension under aseptic conditions. The inoculum was spread uniformly over the leaf surface using a sterile smooth bacteriological loop. All the inoculated leaves were sealed inside transparent plastic bags that contained a piece of wet filter paper so that the RH inside the bags would be 100%. The bags were placed at 22 °C with 15 h light a day for 120 h period. Mock-inoculation treatments were performed as previously using the YMB medium. Pathogen-inoculated (TD) and mock-inoculated (CT) leaves were collected at 24, 72, and 120 HAI; were frozen with liquid nitrogen; and were stored at −80 °C for further processing. Three biological replicates were conducted per treatment (TD and CT), each consisting of a pool of 10 leaves.

### 4.3. Levels of Total Flavonoids and Phenolics on Peach Leaves

An amount of 150 mg of fresh leaf material from all biological replicates of each teatment was grinded with liquid nitrogen and homogenized with 0.1% trichloroacetic acid at 4 °C by vigorous vortexing. Total flavonoid content was assayed as descibed by Tsaniklidis and colleagues [41] using the aluminum chloride colorimetric method with an absorbance at 415 nm, while rutin was used as a standard to make the calibration curve. In addition, the total phenolic content was estimated as described by Chatzistathis and colleagues [42] using the Folin–Ciocalteu colorimetric method, and the absorbance was measured at 750 nm. Gallic acid equivalent was expressed in milligrams per kilogram of fresh mass, with gallic acid serving as the standard reference. The statistical analysis was performed using a parametric one-way ANOVA, and the significance across treatments was inferred using pairwise comparsisons.

### 4.4. Development of a Diagnostic Assay for Pathogen Growth Rate

A novel quantitative real-time PCR (qRT-PCR)-based diagnostic assay was developed to determine the growth rate of *T. deformans* in inoculated peach leaves. A pair of pathogen-specific primers (TdbtqF: 5′-AACGAGCTGGTGGACGGAAAGT-3′ and TdbtqR: 5′-GGTTGTCCCATCGCCAAAAGTC-3′) was designed targeting the partial coding sequences of *T. deformans* beta-tubulin encoding gene. In order to determine the lowest detective threshold, fungal DNA was extracted using the CTAB method from a 4-day-old YMA culture of strain TdLar1 growing at 22 °C. Six serial 10-fold dilutions ranging from 100 ng to 1 pg of fungal DNA were used as PCR templates to determine the sensitivity of the assay and define the detection limit in pure culture. The qRT-PCR was performed on the Applied Biosystems™ StepOne™ Real-Time PCR System (Applied Biosystems, Foster City, CA, USA) using the KAPA SYBR^®^ FAST Universal 2X qPCR Master Mix kit (Kapa Biosystems, Wilmington, MA, USA). The PCR conditions consisted of an initial denaturation at 95 °C for 3 min followed by 40 cycles at 94 °C for 20 s, 60 °C for 20 s, and 72 °C for 1 min, with a final extension step at 72 °C for 3 min. Melting curve analysis were performed over the range of 60 °C to 95 °C to all amplified products. Average cycle threshold (Ct) values of each DNA amount were used to construct a standard curve. 

The species-specific primers (TdbtqF and TdbtqR) were also employed to successfully detect and quantify the fungal DNA in artificially inoculated leaf samples. DNA extractions of artificially pathogen-inoculated (TD) and mock-inoculated leaves (CT) were performed using the CTAB method. For all samples, 10 ng of leaf genomic DNA was equally used as template in 10 μL PCR reactions that were performed as described above. All the assays were repeated using three different biological samples. The quantification data underwent processing using one-way ANOVA. The Tukey test (with a significance level of 99%) was employed to establish minimum significant differences.

### 4.5. Transcriptome Sequencing and Bioinformatics Analysis

Total RNA was extracted from all the 18 leaf samples collected at 24, 72, and 120 HAI across the three biological replicates for each treatment (TD and CT) using the Monarch Total RNA Miniprep Kit (NEB, Frankfurt, Germany). Sequencing libraries were constructed employing the PT042 NGS RNA Library Prep Set (Novogene Ltd.,Cambridge, UK) and then sequenced on the Illumina (San Diego, CA, USA) Novaseq 6000 platform, producing 2 × 150 bp (paired-end) reads. The raw reads were assessed for their quality and sequences containing adapters, and unknown nucleic acids (N) or low-quality reads were removed. Clean reads were selected for further analysis and mapped, employing the HISAT2 software (v. 2.0.5) [43], to the peach reference genomic assembly GCF_000346465.2 of cultivar Lovell [44] obtained from the NCBI database. The feature-counts program [45] was used to count the reads mapped to each gene. Then, the FPKM index was calculated for each gene based on the length of the gene and the number of mapped sequences [46]. The measurements per gene obtained from the mapping were combined using the statistical package of R (v. 4.0.2). The genes were filtered based on their level of expression and by a specific threshold in filtering (cut-off ≥ 10). The DESeq2 R package [47] was used to perform dual comparisons among the data of TD and CT treatments for each time point based on gene expression levels, allowing us to assign DEGs according to an absolute value of log2foldchange ≥ 1, *p*-value ≤ 0.05, and an adjusted *p*-value (padj) ≤ 0.05. The visual representation of differential expression was achieved with the R ggplot package (v. 3.3.5). In addition, Venn diagrams were drawn using the R VennDiagram package (v. 1.7.1). The R clusterProfiler package (v. 3.8.1) was employed to conduct Gene Ontology (GO) enrichment analysis. Gene length bias was corrected [48], and GO terms with a *p*-value < 0.05 were considered to be significantly enriched by DEGs. DEGs were also assigned to KEGG Orthology terms using the KOBAS software v3.0, and the statistical enrichment was tested using the R clusterProfiler package (v. 3.8.1).

### 4.6. Quantitative Real-Time PCR (qRT-PCR) Validation of RNA-Seq Data

The RNA-seq data were validated by qRT-PCR experiments for testing the relative gene expression of nine randomly selected DEGs. First-strand cDNA was synthesized using the LunaScript^®^ RT SuperMix Kit (NEB, Frankfurt, Germany), and the relative gene expression analysis was performed using the Luna^®^ Universal qPCR Master Mix (NEB, Frankfurt, Germany) using three replicates on the QuantStudio^®^ 5 Real-Time PCR System (Applied Biosystems, Foster City, CA, USA). The expression profiles of the nine DEGs were normalized by comparison with the housekeeping gene (LOC18789459, Elongation factor 1-*α*). Relative gene expression ratios of *T. deformans*-inoculated samples compared to mock-inoculated ones were calculated using the 2^−△△CT^ method [49]. 

## 5. Conclusions

Taken together, a dynamic and biphasic transcriptional reprogramming was uncovered in *T. deformans*-inoculated leaves of a susceptible peach cultivar using an RNA-seq approach. The time-dependent expression profiles of specific DEGs may contribute either to the induction of partial defense responses, mainly at 72 HAI and to a less extend at 24 HAI, or to susceptibility, mainly at 120 HAI. Similarly, a diphasic detection of fungal loads in the challenged leaves over the 120 h period was observed with an early maximum peak of fungal DNA quantification detected at 24 HAI followed by a decrease at 72 HAI, turning into a slight rise thereafter at 120 HAI. Therefore, taking into account the dimorphic lifestyle of the fungus (yeast and filamentous forms), *T. deformans* may be more susceptible to host defenses during its early transition to the hyphae biotrophic phase at 72 HAI. These results will be useful in peach breeding programs for disease tolerance against this important pathogen that could result in a significant decrease in fungicides currently used to control *T. deformans*.

## Data Availability

The datasets generated during the current study are available in the NCBI SRA database below: https://www.ncbi.nlm.nih.gov/, PRJNA1071581, created date 31 January 2024.

## Supplementary Materials

The following supporting information can be downloaded at: https://www.mdpi.com/article/10.3390/plants13060861/s1, Figure S1: Bar plots showing the GO enriched terms of DEGs identified across the three comparison groups (TD-24, TD-72, TD-120) related to Biological Process (BP, pink), Cellular Component (CC, green) and Molecular function (MF, blue); Figure S2: Comparison of RNA-seq and RT-qPCR expression values of selected genes in three different time points of peach leaves after *T. deformans* inoculation; Table S1: Summary of RNA-seq data at peach leaves; Table S2: DEGs in peaches leaves during *T. deformans* inoculation at three time points; Table S3: DEGs involved in cell wall modification and modification; Table S4: DEGs involved in pathogen perception and signaling transduction; Table S5: DEGs encoding TFs; Table S6: DEGs encoding pathogenesis-related and defense proteins; Table S7: DEGs involved in secondary and primary metabolism; Table S8: DEGs encoding nutrient and ion transporters; Table S9: Specific primers used in qRT-PCR for the validation of RNA-seq data.

## References

[1] Cimen I., Ertugrul B.B. (2007). Determination of Mycoflora in Almond Plantations under Drought Conditions in Southeastern Anatolia Project Region, Turkey. Plant Pathol. J..

[2] Frisullo S., Rana G.L., Crescenzi A. (2000). Apricot leaf curl in Campania and Apulia (Italy). Petria.

[3] Cissé O.H., Almeida J.M., Fonseca Á., Kumar A.A., Salojärvi J., Overmyer K., Hauser P.M., Pagni M. (2013). Genome sequencing of the plant pathogen *Taphrina deformans*, the causal agent of peach leaf curl. mBio.

[4] Fonseca Á., Rodrigues M.G. (2011). Taphrina fries (1832). The Yeasts.

[5] Rossi V., Bolognesi M., Giosuè S. (2007). Influence of Weather Conditions on Infection of Peach Fruit by *Taphrina deformans*. Phytopathology.

[6] Rodrigues M.G., Fonseca Á. (2003). Molecular Systematics of the Dimorphic Ascomycete Genus Taphrina. Int. J. Syst. Evol. Microbiol..

[7] Svetaz L.A., Bustamante C.A., Goldy C., Rivero N., Müller G.L., Valentini G.H., Fernie A.R., Drincovich M.F., Lara M.V. (2017). Unravelling early events in the *Taphrina deformans*–*Prunus persica* interaction: An insight into the differential responses in resistant and susceptible genotypes. Plant Cell Environ..

[8] Tsai I.J., Tanaka E., Masuya H., Tanaka R., Hirooka Y., Endoh R., Sahashi N., Kikuchi T. (2014). Comparative genomics of Taphrina fungi causing varying degrees of tumorous deformity in plants. Genome Biol. Evol..

[9] Giordani E., Padula G., Radice S. (2013). Compared Anatomy of Young Leaves of *Prunus persica* (L.) Batsch with Different Degrees of Susceptibility to *Taphrina deformans* (Berk.) Tul. J. Phytopathol..

[10] Bassi M., Conti G.G., Barbieri N. (1984). Cell wall degradation by *Taphrina deformans* in host leaf cells: Ultrastructural and cytochemical observations. Mycopathologia.

[11] Doehlemann G., Wahl R., Horst R.J., Voll L.M., Usadel B., Poree F., Stitt M., Pons-Kühnemann J., Sonnewald U., Kahmann R. (2008). Reprogramming a maize plant: Transcriptional and metabolic changes induced by the fungal biotroph *Ustilago maydis*. Plant J..

[12] Ogawa J.M., Zehr E.I., Biggs A.R. (1995). Brown rot. Compendium of Stone Fruit Diseases.

[13] Padula G., Bellini E., Giordani E., Ferri A., Di Vaio C., Damiano C., Fideghelli C. (2008). Further investigations on the resistance to leaf curl (*Taphrina deformans* Berk. Tul.) of peach cultivars and F1 progenies. Atti del VI Convegno Nazionale Sulla Peschicoltura Meridionale.

[14] Nibedita C., Jolly B. (2017). Transcriptomics: A successful approach to unravel the molecular mechanism of plant-pathogen interaction in post-genomic era. Res. J. Biotechnol..

[15] Tyagi P., Singh D., Mathur S., Singh A., Ranjan R. (2022). Upcoming progress of transcriptomics studies on plants: An overview. Front. Plant Sci..

[16] Naidoo S., Visser E.A., Zwart L., du Toit Y., Bhadauria V., Shuey L.S. (2018). Dual RNA-seq to elucidate the plant–pathogen duel. Curr. Issues Mol. Biol..

[17] Li Y.M., Li S.X., Li X.S., Li C.Y. (2018). Transcriptome studies with the third-generation sequencing technology. Life Sci. Instrum..

[18] Agudelo-Romero P., Erban A., Rego C., Carbonell-Bejerano P., Nascimento T., Sousa L., Martínez-Zapater J.M., Kopka J., Fortes A.M. (2015). Transcriptome and metabolome reprogramming in *Vitis vinifera* cv. Trincadeira berries upon infection with *Botrytis cinerea*. J. Exp. Bot..

[19] Liu X., Cao X., Shi S., Zhao N., Li D., Fang P., Chen X., Qi W., Zhang Z. (2018). Comparative RNA-Seq analysis reveals a critical role for brassinosteroids in rose (*Rosa hybrida*) petal defense against *Botrytis cinerea* infection. BMC Genet..

[20] Wang Y., Xiong G., He Z., Yan M., Zou M., Jiang J. (2020). Transcriptome analysis of *Actinidia chinensis* in response to *Botryosphaeria dothidea* infection. PLoS ONE.

[21] Zambounis A., Ganopoulos I., Valasiadis D., Karapetsi L., Madesis P. (2020). RNA sequencing-based transcriptome analysis of kiwifruit infected by *Botrytis cinerea*. Physiol. Mol. Plant Pathol..

[22] AbuQamar S. (2014). Expansins: Cell wall remodeling proteins with a potential function in plant defense. J. Plant Biochem. Physiol..

[23] Yang L., Huang W., Xiong F., Xian Z., Su D., Ren M., Li Z. (2017). Silencing of Sl PL, which encodes a pectate lyase in tomato, confers enhanced fruit firmness, prolonged shelf-life and reduced susceptibility to grey mould. Plant Biotechnol. J..

[24] Macho A.P., Zipfel C. (2014). Plant PRRs and the activation of innate immune signaling. Mol. Cell.

[25] Lannoo N., Van Damme E.J. (2014). Lectin domains at the frontiers of plant defense. Front. Plant Sci..

[26] Delteil A., Gobbato E., Cayrol B., Estevan J., Michel-Romiti C., Dievart A., Kroj T., Morel J.B. (2016). Several wall-associated kinases participate positively and negatively in basal defense against rice blast fungus. BMC Plant Biol..

[27] Haile Z.M., Guzman N.D., Grace E., Moretto M., Sonego P., Engelen K., Moser C., Baraldi E. (2019). Transcriptome profiles of strawberry (*Fragaria vesca*) fruit interacting with *Botrytis cinerea* at different ripening stages. Front. Plant Sci..

[28] De Cremer K., Mathys J., Vos C., Froenicke L., Michelmore R.W., CAMMUE B.P.A., De Coninck B. (2013). RNA seq-based transcriptome analysis of *Lactuca sativa* infected by the fungal necrotroph *Botrytis cinerea*. Plant Cell Environ..

[29] Smith J.E., Mengesha B., Tang H., Mengiste T., Bluhm B.H. (2014). Resistance to *Botrytis cinerea* in *Solanum lycopersicoides* involves widespread transcriptional reprogramming. BMC Genom..

[30] Tsuda K., Somssich I.E. (2015). Transcriptional networks in plant immunity. New Phytol..

[31] Kazan K., Manners J.M. (2013). MYC2: The master in action. Mol. Plant.

[32] Noman A., Aqeel M., Khalid N., Islam W., Sanaullah T., Anwar M., Khan S., Ye W., Lou Y. (2019). Zinc finger protein transcription factors: Integrated line of action for plant antimicrobial activity. Microb. Pathog..

[33] Biswas D., Gain H., Mandal A. (2023). MYB transcription factors in biotic stress tolerance. Plant Stress.

[34] Vargas W.A., Martín J.M., Rech G.E., Rivera L.P., Benito E.P., Díaz-Mínguez J.M., Thon M.R., Sukno S.A. (2012). Plant defense mechanisms are activated during biotrophic and necrotrophic development of *Colletotricum graminicola* in maize. Plant Physiol..

[35] Casañal A., Zander U., Muñoz C., Dupeux F., Luque I., Botella M.A., Schwab W., Valpuesta V., Marquez J.A. (2013). The strawberry pathogenesis-related 10 (PR-10) Fra a proteins control flavonoid biosynthesis by binding to metabolic intermediates. J. Biol. Chem..

[36] Giacomelli L., Zeilmaker T., Giovannini O., Salvagnin U., Masuero D., Franceschi P., Vrhovsek U., Scintilla S., Rouppe Van Der Voort J., Moser C. (2023). Simultaneous editing of two DMR6 genes in grapevine results in reduced susceptibility to downy mildew. Front. Plant Sci..

[37] Petrasch S., Knapp S.J., Van Kan J.A., Blanco-Ulate B. (2019). Grey mould of strawberry, a devastating disease caused by the ubiquitous necrotrophic fungal pathogen *Botrytis cinerea*. Mol. Plant Pathol..

[38] Xiong J.S., Zhu H.Y., Bai Y.B., Liu H., Cheng Z.M. (2018). RNA sequencing-based transcriptome analysis of mature strawberry fruit infected by necrotrophic fungal pathogen *Botrytis cinerea*. Physiol. Mol. Plant Pathol..

[39] Lanver D., Berndt P., Tollot M., Naik V., Vranes M., Warmann T., Münch K., Rössel N., Kahmann R. (2014). Plant surface cues prime *Ustilago maydis* for biotrophic development. PLoS Pathog..

[40] Evans G., Moreno-Rico O., Luna-Ruíz J.J., Sosa-Ramírez J., Moreno-Manzano C.E. (2019). AISLAMIENTO E IDENTIFICACIÓN DE *Taphrina caerulescens* EN Quercus eduardii EN AGUASCALIENTES, MÉXICO. Agrociencia.

[41] Tsaniklidis G., Pappi P., Tsafouros A., Charova S.N., Nikoloudakis N., Roussos P.A., Paschalidis K.A., Delis C. (2020). Polyamine Homeostasis in Tomato Biotic/Abiotic Stress Cross-Tolerance. Gene.

[42] Chatzistathis T., Fanourakis D., Aliniaeifard S., Kotsiras A., Delis C., Tsaniklidis G. (2021). Leaf age-dependent effects of boron toxicity in two *Cucumis melo* varieties. Agronomy.

[43] Kim D., Langmead B., Salzberg S.L. (2015). HISAT: A fast spliced aligner with low memory requirements. Nat. Methods.

[44] Verde I., Abbott A.G., Scalabrin S., Jung S., Shu S., Marroni F., Zhebentyayeva T., Dettori M.T., Grimwood J., International Peach Genome Initiative (2013). The high-quality draft genome of peach (*Prunus persica*) identifies unique patterns of genetic diversity, domestication and genome evolution. Nat. Genet..

[45] Liao Y., Smyth G.K., Shi W. (2014). featureCounts: An efficient general purpose program for assigning sequence reads to genomic features. Bioinformatics.

[46] Mortazavi A., Williams B.A., McCue K., Schaeffer L., Wold B. (2008). Mapping and quantifying mammalian transcriptomes by RNA-Seq. Nat. Methods.

[47] Love M.I., Huber W., Anders S. (2014). Moderated estimation of fold change and dispersion for RNA-seq data with DESeq2. Genome Biol..

[48] Yu G., Wang L.G., Han Y., He Q.Y. (2012). clusterProfiler: An R package for comparing biological themes among gene clusters. Omics J. Integr. Biol..

[49] Livak K.J., Schmittgen T.D. (2001). Analysis of relative gene expression data using real-time quantitative PCR and the 2^−ΔΔCT^ method. Methods.

