# Supplementary material for: Insights into the Transcriptional Reprogramming of Peach Leaves Inoculated with Taphrina deformans
Source: Plants (Basel). 2024 Mar 16;13(6):861. doi: 10.3390/plants13060861 (PMC10976055; doi:10.3390/plants13060861)
Supplement: Supplementary file 1 [file plants-13-00861-s001.zip › Supplementary Figures.pdf]

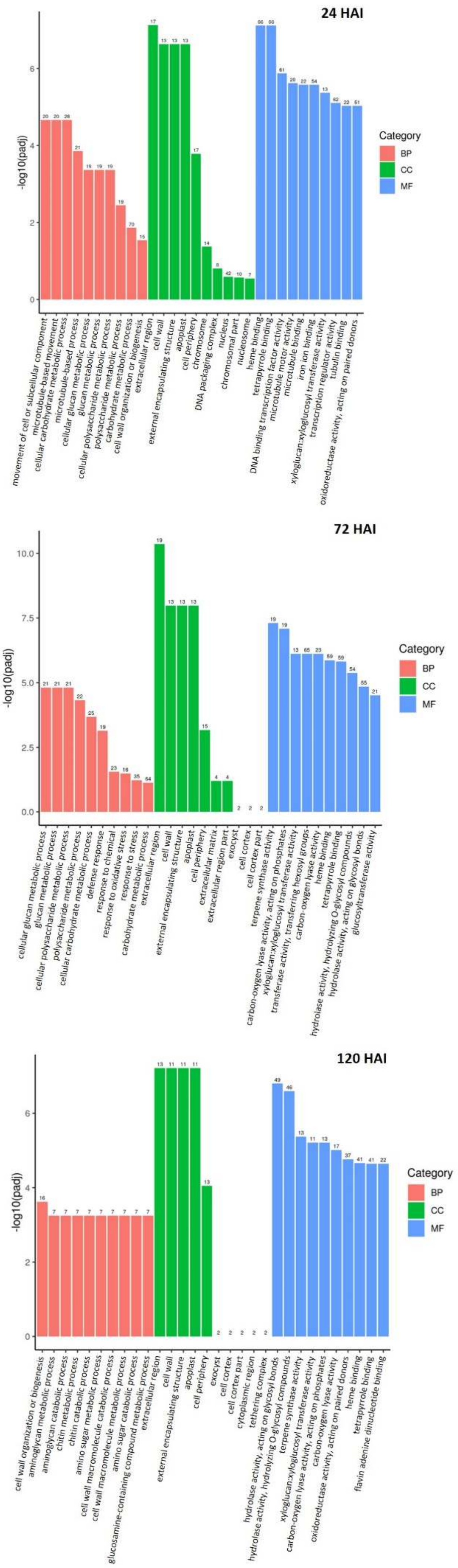

Figure S1. Bar plots showing the GO enriched terms of DEGs identified across the three comparison groups (TD-24, TD-72, TD-120) related to Biological Process (BP, pink), Cellular Component (CC, green) and Molecular function (MF, blue).

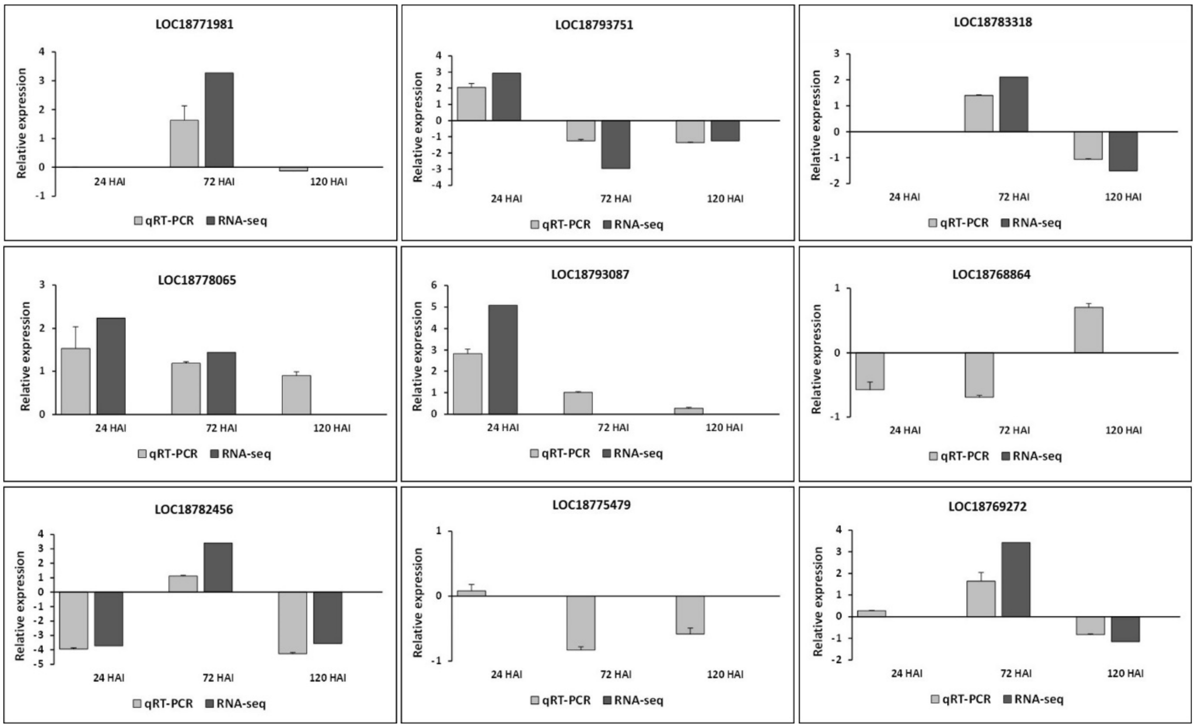

Figure S2. Comparison of RNA-seq and RT-qPCR expression values of selected genes in three different time points of peach leaves after *Taphrina deformans* inoculation. RNA-seq expression levels are depicted as log2foldchange values. RT-qPCR expression levels are represented as mean values from three independent biological replicates.
